# Hypomagnesemia in persons with type 1 diabetes: associations with clinical parameters and oxidative stress

**DOI:** 10.1177/2042018820980240

**Published:** 2020-12-29

**Authors:** Peter R. van Dijk, F. Waanders, Jiedong Qiu, Hannah H. R. de Boer, H. van Goor, H. J. G. Bilo

**Affiliations:** Department of Endocrinology, University Medical Center, University of Groningen, Hanzeplein 1, 9700 RB, Groningen, The Netherlands; Isala, Department of Internal Medicine, Zwolle, The Netherlands; 5th Medical Department, Universitätsklinikum Mannheim, Mannheim, Germany; Department of Endocrinology, University of Groningen, University Medical Center, Groningen, The Netherlands; Department of Pathology and Medical Biology, University of Groningen, University Medical Center, Groningen, The Netherlands; Department of Internal Medicine, University of Groningen, University Medical Center, Groningen, The Netherlands

**Keywords:** glycaemia, insulin, magnesium, oxidative stress, type 1 diabetes

## Abstract

**Background:**

Among persons with type 1 diabetes mellitus (T1DM) low concentrations of magnesium have been reported. Previous (small) studies also suggested a relation of hypomagnesemia with (poor) glycaemic control and complications. We aimed to investigate the magnitude of hypomagnesemia and the associations between magnesium with parameters of routine T1DM care in a population of unselected outpatients.

**Methods:**

As part of a prospective cohort study, initially designed to measure quality of life and oxidative stress, data from 207 patients with a mean age of 45 [standard deviation (SD) 12] years, 58% male, diabetes duration 22 [interquartile range (IQR) 16, 31] years and glycated haemoglobin (HbA1c) of 60 (SD 11) mmol/mol [7.6 (SD 1.0)%] were examined. Hypomagnesemia was defined as a concentration below <0.7 mmol/l.

**Results:**

Mean magnesium concentration was 0.78 (SD 0.05) mmol/l. A deficiency was present in 4.3% of participants. Among these persons, mean concentration was 0.66 (SD 0.03) mmol/l. There was no correlation between magnesium and HbA1c at baseline (*r* = –0.014, *p* = 0.843). In multivariable analysis, free thiols (reflecting the degree of oxidative stress) were significantly and negatively associated with magnesium concentrations.

**Conclusion:**

In this cohort of T1DM outpatients, the presence of hypomagnesemia was infrequent and, if present, relative mild. Magnesium was not associated with glycaemic control nor with presence of micro- and macrovascular complications. Although these results need confirmation, in particular the negative association of magnesium with free thiols, this suggests that hypomagnesemia is not a relevant topic in routine care for people with T1DM.

## Introduction

Magnesium is the second most abundant intracellular and fourth most abundant extracellular cation in the body. It is involved in a wide range of physiologic functions.^1,2^ These functions are achieved through two important properties of magnesium: the ability to form chelates with important intracellular anionic-ligands, especially ATP, and its ability to compete with calcium for binding sites on proteins and membranes. Besides, through dietary intake and subsequent gastro-intestinal absorption, magnesium balance is regulated mainly by equilibrium between renal secretion and reabsorption.

In persons with diabetes, low concentrations of magnesium have been reported in 10–48% of cases.^3,4^ Underlying mechanisms may include increased renal loss through osmotic diuresis or even specific tubular defects.^1,5^ Although insulin does not seem to have an direct effect on magnesium, evidence exists that insulin is able to influence renal reabsorption of magnesium by upregulation of protein expression and activity of the transient receptor potential channel (TRPM) 6 in the distal convoluted tubule.^2,6^ Magnesium supplementation has shown to reduce insulin resistance and improve (short-term) glucose metabolism in both diabetic and non-diabetic subjects.^7,8^ Importantly magnesium, through its effect on inositol transport, has been suggested to be of pathogenic significance in the development of long-term complications of diabetes.^9^ Postulated underlying mechanisms linking magnesium deficiency with complications include higher levels of tumour necrosis factor alpha and increased formation of advances glycosylation end-products.^10,11^

Most studies towards the role of magnesium in diabetes have been performed among persons with type 2 diabetes mellitus (T2DM). Literature on the association of magnesium with parameters of type 1 diabetes (T1DM), including glycaemic control and presence of complications, is scarce. In a recent systemic review and meta-analysis, an association between reduced levels of magnesium and poor glycaemic control was found in T1DM.^11^ However, results were conflicting, with five studies that demonstrated a link between magnesium and glycaemic control while two studies did not. All studies included in the review were cross-sectional, had a modest number of patients (varying from 23 to 138), and focussed mainly on glycaemic control (only five studies assessed complications) and did not provide information about possible underlying mechanisms.

The aim of the present study was to investigate the prevalence of hypomagnesemia in T1DM outpatients and the association of magnesium with clinical parameters, in particular glycated haemoglobin (HbA1c), in order to explore possible underlying factors.

## Patients and methods

This study was designed as a prospective cohort study to investigate several disease factors, including oxidative stress and health-related quality-of-life in persons with T1DM. Full study design and the results of quality-of-life analysis in a subset of patients has been published in detail previously.^12,13^ In brief, from January 1995 to January 1996, consecutive visiting T1DM patients treated at the diabetes outpatient clinic of the Weezenlanden Hospital (nowadays Isala; Zwolle, The Netherlands) were invited to participate in the study. Data concerning demographics, mode of therapy, height, weight, presence of complications, blood pressure and laboratory measurements were collected annually during the study according to a standardised protocol and standardised forms. T1DM was defined as starting insulin therapy within 6 months after the first signs of diabetes and before the age of 30 years, or the absence of C-peptide secretion. In total, 293 patients agreed to participate. In the period from 1996 to 2002, a total of 32 patients dropped out of the study. Reasons for dropping out were: moving out of the area or referral to another physician (*n* = 12), unknown (*n* = 10), lack of interest (*n* = 6), death (*n* = 2) and incorrect diagnosis of T1DM (*n* = 2). For the present analyses, we analysed the 261 patients who were participating in 2002. For 54 patients, no serum samples were available. Therefore, a total of 207 patients were included in the present analysis.

The primary aim of the present study was to investigate the prevalence of hypomagnesemia in T1DM outpatients. As secondary outcomes, the cross-sectional associations between baseline magnesium concentrations with indices of T1DM management, including HbA1c and the presence of complications, were assessed.

Participants were instructed to be in a fasting state when the blood samples were collected. Venous blood samples were collected into BD Vacutainer™ serum tubes, centrifuged and the serum was stored directly in aliquots at −80°C (without thawing) until measurement. Serum magnesium was measured on a Modular Analytics P-module (Roche Diagnostics, Mannheim, Germany). Low-density lipoprotein (LDL) cholesterol was quantitated indirectly using the Friedewald formula. A magnesium deficiency was defined as serum concentration <0.7 mmol/l.^14,15^ HbA1c was measured using affinity chromatography high-performance liquid chromatography (Ultra 2, Trinity Biotech, Kansas City, MO, USA). In order to assess systemic redox status, we measured the concentration of serum free thiols.^16^ Serum-free thiols are compounds with a free sulfhydryl (R–SH) group, and are oxidized readily by reactive oxygen species. In previous studies, in a variety of diseases, R–SH have been linked with oxidative stress and clinical outcome.^17^ R–SH were measured as previously described, with minor modifications.^18^

Macrovascular complications were defined as angina pectoris (AP), peripheral artery disease (PAD), myocardial infarction (MI), percutaneous transluminal coronary angioplasty (PTCA), coronary artery bypass grafting (CABG), cerebrovascular accident (CVA) or transient ischemic attack (TIA). Microvascular complications were defined as diabetic retinopathy, albuminuria (both micro- and macroalbuminuria) and diabetic peripheral neuropathy. For more detailed definitions of these microvascular complications, we refer to a previous publication.^19^

Descriptive summaries included the mean with standard deviation (SD) for normally distributed variables and the median with the interquartile range (IQR; 25th–75th percentile) for non-normally distributed variables. Normality was checked with normality plots. For categorical variables, absolute number of patients (%) for each magnesium tertile is shown. One-way ANOVA test was used to analyse different magnesium tertiles on continuous parameters. Approximately normal distribution in each tertile was tested using normality plots beforehand. Homogeneity of variances was tested using the Levene’s test and Welch-correction applied if necessary. In case of a non-normal distribution, non-parametric Kruskal–Wallis test was conducted instead. Chi-square test and Fisher’s exact test for *p* value due to the low number of events was performed to analyse different magnesium tertiles on categorical parameters.

Univariable analysis for correlation, using the Pearson product-moment correlation coefficient for continuous data and point-biserial correlation coefficient for categorical data, was performed to investigate the association between serum magnesium and other variables. Linearity was checked on a scatter plot. Extreme outliers defined as 3 × IQR from the 25th or 75th percentile were identified on a box-whisker blot and excluded if it was a multivariate outlier. Non-parametric Spearman’s rank order correlation was performed for non-normally distributed parameters. Next, multivariable linear regression analysis (method: forced-entry) was performed to investigate associations between serum magnesium as dependent variable and multiple independent variables. Variables were used in the multivariable model based on previous literature [HbA1c, serum creatinine, R–SH, high-density lipoprotein (HDL) cholesterol, LDL cholesterol, triglycerides and the presence of microvascular complications]^11^ or in case the *p* value was ⩽0.1 in the univariable analysis. The model was checked for collinearity and, for variables with a non-parametric distribution, natural logarithmic (ln) transformation was performed. The quality of the model was described using the accuracy of the variance prediction by the adjusted *R*^2^ value. Normality and homoscedasticity of residuals are checked with normality plots and scatter plot. A (two-sided) *p* value of less than 0.05 was considered statistically significant. All analyses were performed using SPSS version 25.0 (SPSS Inc, Chicago, IL, USA). A (two-sided) *p* value of less than 0.05 was considered statistically significant. The study was performed in accordance with the Declaration of Helsinki. Written and oral informed consent was obtained from all patients, and the local medical ethics committee of Isala (METC Isala, Zwolle, the Netherlands) approved the protocol.

## Results

Baseline characteristics of the 207 participants are presented in Table 1. Mean age of the cohort was 45.4 (SD 11.7) years, 58% was male, diabetes duration 22.4 (IQR 16.0, 30.9) years, baseline HbA1c was 7.6 (SD 1.0) % [60.0 (SD 11.2) mmol/mol]: 106 (51.2%) persons had a microvascular complication and 22 (10.6%) a macrovascular complication.

**Table 1.** Baseline characteristics of all patients and per tertile of Mg.

	All	Tertiles of magnesium		
	*n* = 207	Low (0.60–0.75 mmol/l) *n* = 69	Middle (0.75–0.80 mmol/l) *n* = 69	High (0.80–0.93 mmol/l) *n* = 69
**Magnesium (mmol/l)**	0.78 (0.05)	0.73 (0.02)	0.78 (0.01)	0.84 (0.03)
**Demographics**				
Age (years)	45.4 (11.7)	47.4 (12.2)	44.7 (11.9)	44.1 (10.9)
Diabetes duration (years)	21.8 (15.8, 29.1)	21.2 (14.1, 28.3)	22.1 (16.3, 32.2)	22.7 (15.4, 29.0)
Male gender (*n*)	120 (58.0%)	38 (55.1%)	42 (60.9%)	40 (58.0%)
BMI (kg/m^2^)	25.4 (23.3, 28.4)	24.9 (23.1, 28.0)	25.3 (23.2, 27.8)	26.5 (23.4, 29.6)
Current smoker (yes)	47 (27.6%)	16 (23.2%)	18 (26.1%)	13 (18.8%)
Systolic blood pressure (mmHg)	130.4 (17.9)	132.6 (18.8)	130.4 (17.2)	127.9 (17.6)
Diastolic blood pressure (mmHg)	77.1 (10.2)	78.4 (11.0)	77.4 (8.7)	75.3 (10.7)
Mode of insulin administration: MDI	119 (57.5%)	43 (62.3%)	40 (58.0%)	36 (52.2%)
Mode of insulin administration: CSII	88 (42.5%)	26 (37.7%)	29 (42.0%)	33 (47.8%)
Total insulin dose^a^	56.8 (19.6)	48.0 (35.0, 62.0)	60.0 (42.0, 66.0)	68.0 (54.0, 88.0)
**Complications**				
Microvascular complications				
Retinopathy (*n*)	88 (42.5%)	31 (44.9%)	25 (76.8%)	32 (46.4%)
Neuropathy (*n*)	24 (11.6%)	11 (15.9%)	8 (11.6%)	5 (7.2%)
Micro-albuminuria	28 (13.5%)	8 (11.6%)	13 (18.8%)	7 (10.1%)
Macro-albuminuria	8 (3.9%)	1 (1.4%)	3 (4.3%)	4 (5.8%)
Macrovascular complications				
Angina pectoris (*n*)	3 (1.4%)	0 (0.0%)	1 (1.4%)	2 (2.9%)
MI (*n*)	3 (1.4%)	0 (0.0%)	1 (1.4%)	2 (2.9%)
PTCA (*n*)	3 (1.4%)	2 (2.9%)	1 (1.4%)	0 (0.0%)
PAD (*n*)	5 (2.3%)	1 (1.4%)	2 (2.9%)	2 (2.9%)
CABG (*n*)	3 (1.4%)	2 (2.9%)	1 (1.4%)	0 (0.0%)
TIA (*n*)	2 (1.0%)	0 (0.0%)	1 (1.4%)	1 (1.4%)
CVA (*n*)	4 (1.9%)	1 (1.4%)	1 (1.4%)	2 (2.9%)
**Laboratory measurements**				
HbA1c (%)	7.6 (1.0)	7.7 (1.1)	7.6 (1.1)	7.7 (1.0)
HbA1c (mmol/mol)	60.0 (11.2)	60.3 (11.5)	59.0 (11.6)	60.7 (10.9)
Creatinine (µmol/l)	84.5 (77.3, 93.8)	84.0 (76.0, 93.0)	84.0 (77.0, 93.0)	86.0 (79.0, 95.0)
eGFR (MDRD, ml/min/1.73 m^2^)	126.2 (109.6, 141.8)	124.6 (109.0, 138.7)	124.6 (108.9, 141.3)	129.4 (111.3, 146.0)
Total cholesterol (mmol/l)	4.6 (1.0)	4.6 (1.0)	4.6 (0.9)	4.7 (1.1)
HDL cholesterol (mmol/l)	1.5 (0.4)	1.6 (0.4)	1.5 (0.4)	1.5 (0.4)
Total cholesterol:HDL ratio	4.5 (4.0, 5.2)	2.9 (2.3, 3.7)	3.1 (2.5, 3.6)	3.1 (2.6, 4.0)
LDL cholesterol (mmol/l)	2.6 (0.9)	2.5 (0.9)	2.5 (0.8)	2.7 (1.0)
Triglycerides (mmol/l)	0.9 (0.7, 1.4)	1.0 (0.6, 1.2)	1.0 (0.6, 1.5)	0.9 (0.7, 1.4)
C-reactive protein (mg/l)	2.0 (1.0, 3.0)	1.0 (1.0, 4.0)	1.0 (1.0, 3.0)	2.0 (1.0, 3.0)
R–SH (µM)	285.0 (34.6)	282.6 (36.4)	282.3 (33.2)	280.4 (34.6)
NT-proBNP (pmol/l)	40.5 (16.2, 86.2)	47.4 (19.7, 110.1)	29.7 (11.0, 70.1)	49.4 (19.7, 86.0)
Calcium (mmol/l)	2.29 (2.20, 2.35)	2.28 (2.21, 2.33)	2.30 (2.23, 2.38)	2.30 (2.24, 2.36)
Albumin (g/l)	42.2 (2.6)	41.5 (2.5)	42.4 (2.5)	42.7 (2.8)
Phosphate (mmol/l)	1.0 (0.8, 1.1)	0.9 (0.8, 1.1)	1.0 (0.8, 1.1)	1.0 (0.8, 1.1)
PTH (pmol/l)	2.8 (2.3, 3.6)	2.8 (2.5, 3.5)	2.7 (2.2, 3.8)	2.8 (2.3, 3.4)
25 OH vit D	51.4 (37.2, 72.2)	46.6 (36.3, 72.8)	50.3 (36.0, 70.2)	54.3 (41.7, 73.2)

Data are presented as number (%), mean (SD) or median (IQR).

a *n* = 29.

BMI, body mass index; CABG, coronary artery bypass grafting; CSII, continuous subcutaneous insulin infusion; CVA, cerebral vascular event; eGFR, estimated glomerular filtration rate; HbA1c, glycated haemoglobin A1c; HDL, high-density lipoprotein; IQR, interquartile range; LDL, low-density lipoprotein; MDI, multiple daily injections; MDRD, modification of diet in renal disease; MI, myocardial infarction; NA, not applicable; NT-proBNP, N-terminal pro-B type natriuretic peptide; PTCA, percutaneous transluminal coronary angioplasty; PTH; parathyroid hormone; R–SH, total free thiol groups; SD, standard deviation; TIA, transient ischemic attack.

Baseline magnesium concentration was distributed normally with a mean of 0.78 (SD 0.05) mmol/l. There were no differences in baseline characteristics between the tertiles of magnesium (see Table 1, all *p* values > 0.05). A magnesium deficiency (i.e. serum concentration <0.7 mmol/l) was present in nine (4.3%) participants; among them the magnesium concentration was 0.66 (SD 0.03) mmol/l with a range of 0.60–0.69 mmol/l. Magnesium concentrations did not differ between men and woman [0.78 (SD 0.06) mmol/l *versus* 0.78 (SD 0.05) mmol/l, *p* = 0.58]. There was no significant correlation between magnesium and HbA1c at baseline (*r* = –0.014, *p* = 0.843). In multivariable regression, concentrations of albumin and R–SH were significantly associated with magnesium concentrations (Table 2).

**Table 2.** Univariate and multivariate linear regression analysis for association with magnesium.

	Univariable		Multivariable	
	Correlation coefficient	*p* value	St. Beta	*p* value
**Demographics**				
Age (years)	−0.07	0.328		
Diabetes duration (years)	0.02	0.813		
Male gender (*n*)	0.04	0.580		
BMI (kg/m^2^)	0.04	0.581		
Current smoker (yes)	−0.08	0.306		
Systolic blood pressure (mmHg)	−0.03	0.674		
Diastolic blood pressure (mmHg)	−0.09	0.236		
Mode of insulin administration: MDI (*n*)	0.01	0.940		
Mode of insulin administration: CSII (*n*)	−0.01	0.940		
Total insulin dose (IE)^a^	**0.45**	**0.014**	*n* too small	
**Complications**				
*Microvascular complications*				
Retinopathy (*n*)	0.08	0.286		
Neuropathy (*n*)	−0.12	0.122		
Micro-albuminuria (*n*)	0.03	0.712		
Macro-albuminuria (*n*)	0.07	0.303		
*Macrovascular events*				
Angina pectoris (*n*)	0.05	0.507		
MI (*n*)	0.12	0.099		
PTCA (*n*)	−0.05	0.449		
PAD (*n*)	0.03	0.663		
CABG (*n*)	−0.12	0.081	−0.103	0.142
TIA (*n*)	0.04	0.589		
CVA (*n*)	0.03	0.629		
**Laboratory measurements**				
HbA1c (%)	−0.01	0.843	−0.094	0.195
HbA1c (mmol/mol)	−0.01	0.843		
Creatinine (µmol/l)	0.08	0.276	0.104^b^	0.148
eGFR (MDRD, ml/min/1.73 m^2^)	0.11	0.110		
Total cholesterol (mmol/l)	0.08	0.272	0.154	0.766
HDL cholesterol (mmol/l)	−0.10	0.165	−0.154	0.514
Total cholesterol:HDL ratio	0.12	0.084		
LDL cholesterol (mmol/l)	0.11	0.118	−0.042	0.927
Triglycerides (mmol/l)	0.05	0.503	−0.094^b^	0.612
C-reactive protein (mg/l)	−0.05	0.516		
R–SH (µM)	−0.06	0.416	**–0.213**	**0.007**
NT-proBNP (pmol/l)	−0.01	0.886		
Calcium (mmol/l)	0.08	0.230		
Albumin (g/l)	**0.19**	**0.007**	**0.298**	**0.000**
Phosphate (mmol/l)	0.06	0.412		
PTH (pmol/l)	0.01	0.930		
25-OH Vitamin D (ng/ml)	−0.05	0.462		

Adjusted *R*² for the multivariable model: 0.075; *F* (9, 189) = 2.79, *p* = 0.004.

a *n *= 29.

b St.Beta of the ln-transformed parameter.

BMI, body mass index; CABG, coronary artery bypass grafting; CSII, continuous subcutaneous insulin infusion; CVA, cerebral vascular event; eGFR, estimated glomerular filtration rate; HbA1c, glycated haemoglobin A1c; HDL, high-density lipoprotein; LDL, low-density lipoprotein; MDI, multiple daily injections; MDRD, modification of diet in renal disease; MI, myocardial infarction; NT-proBNP, N-terminal pro-B type natriuretic peptide; PTCA, percutaneous transluminal coronary angioplasty; PTH; parathyroid hormone; R–SH, total free thiol groups; TIA, transient ischemic attack.

## Discussion

In the current cohort of outpatients with T1DM, hypomagnesemia (defined as a magnesium concentration below <0.7 mmol/l) was present in only 4.3% of participants. Amongst these persons, the magnitude of the hypomagnesemia was relatively mild. In multivariate analysis, magnesium concentrations were associated with albumin and R–SH (a marker of systemic oxidative stress), but not with HbA1c nor the presence of complications.

The results of this study are to some degree in conflict with previous literature. First of all, only a low number of patients in the present study had hypomagnesemia. In previous studies, the percentage of hypomagnesemia among persons with T1DM has been reported to be up to 38.6%.^20–22^ Of note, differences in age categories (children and adolescents)^20,22^ and definitions of hypomagnesemia (varying from <0.75 mmol/l to <2 SDs) hamper proper comparisons.^21,22^ Nevertheless, based upon our data, the prevalence and magnitude of hypomagnesemia amongst Dutch persons with T1DM seems to be modest. The present study included an unselected population of outpatients with a rather normal renal function but some signs of renal damage as exemplified by the presence of albuminuria; given the pivotal role of the kidney in magnesium metabolism, this might explain the low prevalence of lack of hypomagnesemia in the present population.

Secondly, we did not demonstrate an association between magnesium and HbA1c levels. In a recent meta-analysis of previous literature, five of the seven studies that were designed to evaluate the relation between magnesium and glycaemic control showed an association between reduced levels of magnesium and measures of glycaemia. It should be taken into account that considerable differences in the method of measuring magnesium (colorimetric, spectrophotometry by atomic absorption and selective ion electrode), measures of glycaemia (HbA1c, glucose and fructosamine) and average degree of glycaemic control and presence of complications were present in these studies. Notably, there does not seem to be a clear (patho)physiologic influence of glucose and insulin on magnesium metabolism. To this extent, given the small number of persons with available data for daily insulin dose, *n* = 29), the association between insulin dose and magnesium found in the current study should be interpreted with caution.

We find a significant negative relation between magnesium and R–SH, a marker of systemic oxidative stress. The plasma concentrations of total R–SH are proposed to reflect the systemic body redox status, indicating that a decline in circulating R–SH reveals enhancement of the oxidative tone. The negative association between magnesium and R–SH is therefore surprising, as magnesium depletion has previously been associated with increased oxidative stress.^23^ Given the fact that this study was not designed nor powered to investigate the association between magnesium and R–SH, our finding should be interpreted with caution. It certainly needs confirmation in a properly powered study.

In cross-sectional analysis, we were unable to demonstrate a relation between micro- and macrovascular complications and magnesium. Previously, the presence of diabetic nephropathy (albuminuria) and retinopathy was found to be associated (in cross-sectional analyses) with low magnesium concentrations.^20,24^ However, in accordance with our results, (most) other studies did not find an association.^22,25–27^

Strengths of the study include the sample size and the characterisation of the population. Although the present study has the largest sample size to date in literature, this sample is still modest. The lack of a non-diabetic reference population, the limited confirmation of T1DM diagnosis at the start of the study (also reflected by re-diagnosis during follow-up in two participants), amount of drop-out of the original cohort and the lack of other plasma antioxidant species such as ascorbate, uric acid and small-molecular-weight thiols and other markers of systemic oxidative stress should be mentioned. Another important limitation of the present study is the lack of information concerning use of medication including (over the counter) magnesium supplements and proton pump inhibitors. Furthermore, missing data including insulin dose and non-diabetes related causes of low magnesium concentrations such as decreased intake or malabsorption, or increased gastro-intestinal loss, for example, due to diarrhoea or medication, cannot be excluded in the present cohort. Therefore, the results of our study need confirmation and should be interpreted with caution.

In conclusion, hypomagnesemia was infrequently (4.3%) present in outpatients with T1DM. Concentrations of magnesium were significantly associated with a marker of systemic oxidative stress in persons with T1DM. Despite that, no association with baseline glycaemic control or complications were present. Although this study needs confirmation, it seems that hypomagnesemia is currently not a relevant topic in routine care for T1DM outpatients.
